# Androgen deprivation therapy and excess mortality in men with prostate cancer during the initial phase of the COVID-19 pandemic

**DOI:** 10.1371/journal.pone.0255966

**Published:** 2021-10-07

**Authors:** Rolf Gedeborg, Johan Styrke, Stacy Loeb, Hans Garmo, Pär Stattin

**Affiliations:** 1 Department of Surgical Sciences, Uppsala University, Uppsala, Sweden; 2 Department of Surgical and Perioperative Sciences, Urology and Andrology, Umeå University, Umeå, Sweden; 3 Department of Urology and Population Health, New York University and Manhattan Veterans Affairs Medical Center, New York, New York; 4 Translational Oncology and Urology Research (TOUR), King’s College London, Guy’s Hospital, London, United Kingdom; Medizinische Universitat Innsbruck, AUSTRIA

## Abstract

**Background:**

Men have a higher risk of death from COVID-19 than women and androgens facilitate entrance of the SARS-CoV-2 virus into respiratory epithelial cells. Thus, androgen deprivation therapy may reduce infection rates and improve outcomes for COVID-19. In the spring of 2020, Sweden was highly affected by COVID-19. The aim was to estimate the impact of androgen deprivation therapy on mortality from COVID-19 in men with prevalent prostate cancer by comparing all-cause mortality in the spring of 2020 to that in previous years.

**Patients and methods:**

Using the Prostate Cancer data Base Sweden all men with prostate cancer on March 1 each year in 2015–2020 were followed until June 30 the same year. Exposure to androgen deprivation therapy was ascertained from filled prescriptions for bicalutamide monotherapy, gonadotropin-releasing hormone agonists (GnRH), or bilateral orchidectomy.

**Results:**

A total of 9,822 men died in March-June in the years 2015–2020, of whom 5,034 men were on androgen deprivation therapy. There was an excess mortality in 2020 vs previous years in all men. The crude relative mortality rate ratio for 2020 vs 2015–2019 was 0.93 (95% confidence interval (CI) 0.83 to 1.04) in men on GnRH, and 0.90 (95% CI 0.78 to 1.05) in men on bicalutamide monotherapy. After multivariable adjustment these ratios were attenuated to 1.00 (95% CI 0.89 to 1.12) and 0.97 (95% CI 0.84 to 1.12), respectively. When restricting the analysis to the regions with the highest incidence of COVID-19 or to the time period between 2 April to 10 June when mortality in 2020 was increased >30% compared to previous years, the results were similar to the main analysis.

**Conclusions:**

In this large national population-based cohort of men with prevalent prostate cancer, there was no clear evidence in support for an effect of androgen deprivation therapy on COVID-19 mortality.

## Introduction

Men have consistently been shown to have approximately 50% higher risk of COVID-19 compared to women, and the increase in risk remained after adjustment for risk factors [1, 2]. A similarly increased risk in men has also been observed for critical care admission and death in COVID-19 [2–5]. These differences associated with sex have also been observed in children [6, 7].

One proposed mechanism for the susceptibility of men to COVID-19 is that androgen signaling facilitates entry of the virus into host cells [8]. Internalization of the SARS-CoV-2 virus relies on proteases such as the Transmembrane Protease Serine 2 (TMPRSS2) surface protein expressed by epithelial cells (e.g in the lung). *In vitro*, inhibition of the TMPRSS2 protease activity resulted in decreased entry of SARS-CoV-2 into pulmonary epithelial cells [8]. The regulation of TMPRSS2 expression is androgen-dependent, suggesting that androgen deprivation therapy (ADT) could be protective against COVID-19 [9]. The response to gonadotropin-releasing hormone (GnRH) agonists would then from a mechanistic perspective be of primary interest. While this is an interesting hypothesis, further support from patient-relevant clinical outcomes is needed. An early study reported that men with prostate cancer not on ADT had a four-fold increased risk of COVID-19 and a more severe disease compared with men on ADT [10]. However, the results in this study were based on 118 men with COVID-19 of whom merely four were on ADT and 114 were not. Two subsequent small studies failed to replicate this association [11, 12]. Based on these findings, several clinical trials have been initiated to evaluate the benefit of ADT in men with COVID-19 [13]. However, in men with prostate cancer, use of ADT has been associated with adverse effects including an increased risk of cardiovascular disease [14] even within six months after initiation, and this increase has been particularly strong in men with prior cardiovascular disease [15]. Men hospitalized with COVID-19 often have cardiovascular risk factors [16], but it is unknown if very short-term ADT for COVID-19 affects cardiovascular risk. There are also numerous other potential mechanisms that could lead to the observed male susceptibility to COVID-19 [17].

We took advantage of a unique setting in which we had access to diagnosis, treatment and vital status up to July 2020 for virtually all men with prostate cancer in Sweden, with a source population of more than 10 million people that was strongly affected by the COVID-19 pandemic in the spring of 2020.

The aim of this study was to investigate if hormonal treatment for prostate cancer is associated with a reduction of mortality risk related to COVID-19.

## Patients and methods

### Data source

Men with prostate cancer were identified in The National Prostate Cancer Register (NPCR) of Sweden, which since 1998 captures 98% of all cases of prostate cancer in Sweden as compared to the Swedish Cancer Register to which registration is mandatory. NPCR contains comprehensive data on cancer characteristics and primary treatment [18].

By use of the Personal Identity Number unique for every Swedish citizen, data for men in NPCR diagnosed up to 31 Dec, 2019 was linked in July 2020 (i.e. with follow-up on vital status until 1 July 2020) to the Cancer Register [19], statistics on deaths submitted by the Swedish Tax Agency to Statistics Sweden, the Prescribed Drug Register [20], and the National Patient Register [21] held at The National Board of Health and Welfare [22], to create the Prostate Cancer data Base Sweden (PCBaSe) RAPID 2019. The study was approved by the Ethical Review Authority. Since this was a study exclusively based on existing large-scale registry data the requirement for individual informed consent was waived by the Authority.

### Study population and study period

In PCBaSe an open cohort was defined with men with prevalent prostate cancer alive on 1 March 2020 and similar subsets for corresponding months in 2015–2019. Men from three regions in which GnRH agonists were provided directly from the hospital without prescription were excluded. The remaining 18 Swedish regions forming the source population for the study covered 91% of the Swedish population as of 31 December 2019. For complementary analyses, a more restricted time interval was defined from the first to the last date in 2020 with a mortality rate more than 30% above the average mortality rate in 2015–2019.

Separate analyses were also performed within the ten regions with the highest cumulative number of cases of COVID-19 as determined on 28 June 2020 from data published by the Public Health Agency of Sweden (**S1 Fig**).

### Exposure

Exposure to ADT was ascertained from fillings in The Prescribed Drug Register for bicalutamide monotherapy (Anatomical Therapeutic Chemical (ATC) code L02BB03 and also the rare use (~2%) of flutamide ATC code L02BB01), gonadotropin-releasing hormone (GnRH) agonists (L02AE). We have previously demonstrated a very high adherence to ADT and that it is rare that a man prescribed GnRH agonists does not continue his medication [23–27]. Men who in addition to GnRH agonist received 30 days of flare prophylaxis with bicalutamide were classified as exposed to GnRH agonist only. Data on bilateral orchidectomy (procedure code KFC10 or KFC15) were extracted from the National Patient Register.

### Outcome

The number of deaths per day from all causes was counted by use of the date of death. These counts were used to compare mortality in 1 March to 30 June in 2020 to corresponding months in 2015–2019. Mortality for the entire Swedish population during these time periods is also provided to contextualize the results.

### Covariates

Age at death (stratified), county of residence, prostate cancer risk category (absence/presence of distant metastases at the date of prostate cancer diagnosis), history of curative treatment (radical prostatectomy or radiotherapy), the Charlson Comorbidity Index and a Drug Comorbidity Index [28] were used as covariates in regression models. The Charlson Comorbidity Index was calculated from hospital discharge diagnoses registered in the National Patient Register during the 10-year period prior to start of follow-up [29, 30]. The Drug Comorbidity Index was calculated from filled prescriptions during a 365-day interval preceding the start of follow-up. Prescribed medications were categorized by chemical subgroup, i.e. the first five positions of the ATC code [28, 31]. Only ATC codes prevalent in at least 1% of observed deaths were used to calculate the Drug Comorbidity Index. Calendar year and day in the study period were also used as covariates to generate more comparable time periods.

### Statistics

The mortality rates by calendar day for each exposure group and calendar time period were calculated and plotted with locally weighted smoothing (LOESS). Using the log of numbers at risk as offset in a Poisson regression the rate of death was compared between 2020 and the average from previous years. Complementary analyses were performed in which the analyses were restricted to the 10 regions with the highest incidence of COVID-19, and the calendar period between 2 April to 10 June when excess mortality was above 30%.

## Results

Baseline characteristics and exposure for study men are described in **Table 1**.

**Table 1:** Baseline characteristics.

	2015		2016		2017		2018		2019		2020	
	(*N* = 86,400)^a^		(*N* = 91,403)^a^		(*N* = 96,212)^a^		(*N* = 100,712)^a^		(*N* = 105,679)^a^		(*N* = 110,371)^a^	
**Age**, *N* (%)												
≤60 years	4,507	(5.2)	4,620	(5.1)	4,788	(5.0)	4,852	(4.8)	4,968	(4.7)	5,072	(4.6)
61–70 years	24,556	(28.4)	24,569	(26.9)	24,384	(25.3)	24,121	(24.0)	24,028	(22.7)	23,939	(21.7)
71–75 years	20,541	(23.8)	22,792	(24.9)	24,430	(25.4)	25,484	(25.3)	25,917	(24.5)	25,818	(23.4)
76–80 years	16,680	(19.3)	17,882	(19.6)	19,510	(20.3)	21,511	(21.4)	23,965	(22.7)	26,463	(24.0)
>80 years	20,116	(23.3)	21,540	(23.6)	23,100	(24.0)	24,744	(24.6)	26,801	(25.4)	29,079	(26.3)
**Charlson Comorbidity Index**, N (%)												
0	53,476	(61.9)	56,373	(61.7)	59,267	(61.6)	61,887	(61.4)	64,883	(61.4)	68,317	(61.9)
1	14,649	(17.0)	15,564	(17.0)	16,324	(17.0)	17,067	(16.9)	17,841	(16.9)	18,599	(16.9)
2	10,837	(12.5)	11,574	(12.7)	12,356	(12.8)	13,051	(13.0)	13,692	(13.0)	14,048	(12.7)
3+	7,438	(8.6)	7,892	(8.6)	8,265	(8.6)	8,707	(8.6)	9,263	(8.8)	9,407	(8.5)
**Drug Comorbidity Index**, *N* (%)												
≤0	14,583	(16.9)	15,496	(17.0)	15,862	(16.5)	16,618	(16.5)	17,595	(16.6)	18,193	(16.5)
0–1	27,207	(31.5)	28,621	(31.3)	30,199	(31.4)	31,646	(31.4)	33,132	(31.4)	34,485	(31.2)
1–2	17,697	(20.5)	18,598	(20.3)	19,640	(20.4)	20,686	(20.5)	21,768	(20.6)	22,793	(20.7)
2–4	17,655	(20.4)	18,778	(20.5)	19,856	(20.6)	20,832	(20.7)	21,825	(20.7)	22,983	(20.8)
4+	9,258	(10.7)	9,910	(10.8)	10,655	(11.1)	10,930	(10.9)	11,359	(10.7)	11,917	(10.8)
**Androgen deprivation therapy**, *N* (%)												
GnRH	11,313	(13.1)	11,329	(12.4)	11,278	(11.7)	10,831	(10.8)	10,734	(10.2)	10,555	(9.6)
Bicalutamide monotherapy	8,656	(10.0)	9,588	(10.5)	10,537	(11.0)	11,427	(11.3)	12,432	(11.8)	13,172	(11.9)
None	66,431	(76.9)	70,486	(77.1)	74,397	(77.3)	78,454	(77.9)	82,513	(78.1)	86,644	(78.5)
**Curative therapy**, *N* (%)												
No	39,670	(45.9)	40,746	(44.6)	41,716	(43.4)	42,559	(42.3)	43,846	(41.5)	45,640	(41.4)
Yes	46,730	(54.1)	50,657	(55.4)	54,496	(56.6)	58,153	(57.7)	61,833	(58.5)	64,731	(58.6)
**Distant metastases at diagnosis**, *N* (%)												
No	82,163	(95.1)	86,971	(95.2)	91,612	(95.2)	95,958	(95.3)	100,779	(95.4)	105,324	(95.4)
Yes	4,237	(4.9)	4,432	(4.8)	4,600	(4.8)	4,754	(4.7)	4,900	(4.6)	5,047	(4.6)
**County with high COVID-19 incidence**^**b**^, *N* (%)												
No	39,531	(45.8)	41,942	(45.9)	44,248	(46.0)	46,565	(46.2)	48,882	(46.3)	50,917	(46.1)
Yes	46,869	(54.2)	49,461	(54.1)	51,964	(54.0)	54,147	(53.8)	56,797	(53.7)	59,454	(53.9)

^a^ Note that the same individual may be present in multiple columns.

^b^ See online-only supplemental material for definition.

Comparison of baseline characteristics at start of follow up on 1 March each respective year for men with prevalent prostate cancer in Prostate Cancer data Base Sweden (PCBaSe) RAPID 2019.

**Fig 1:**
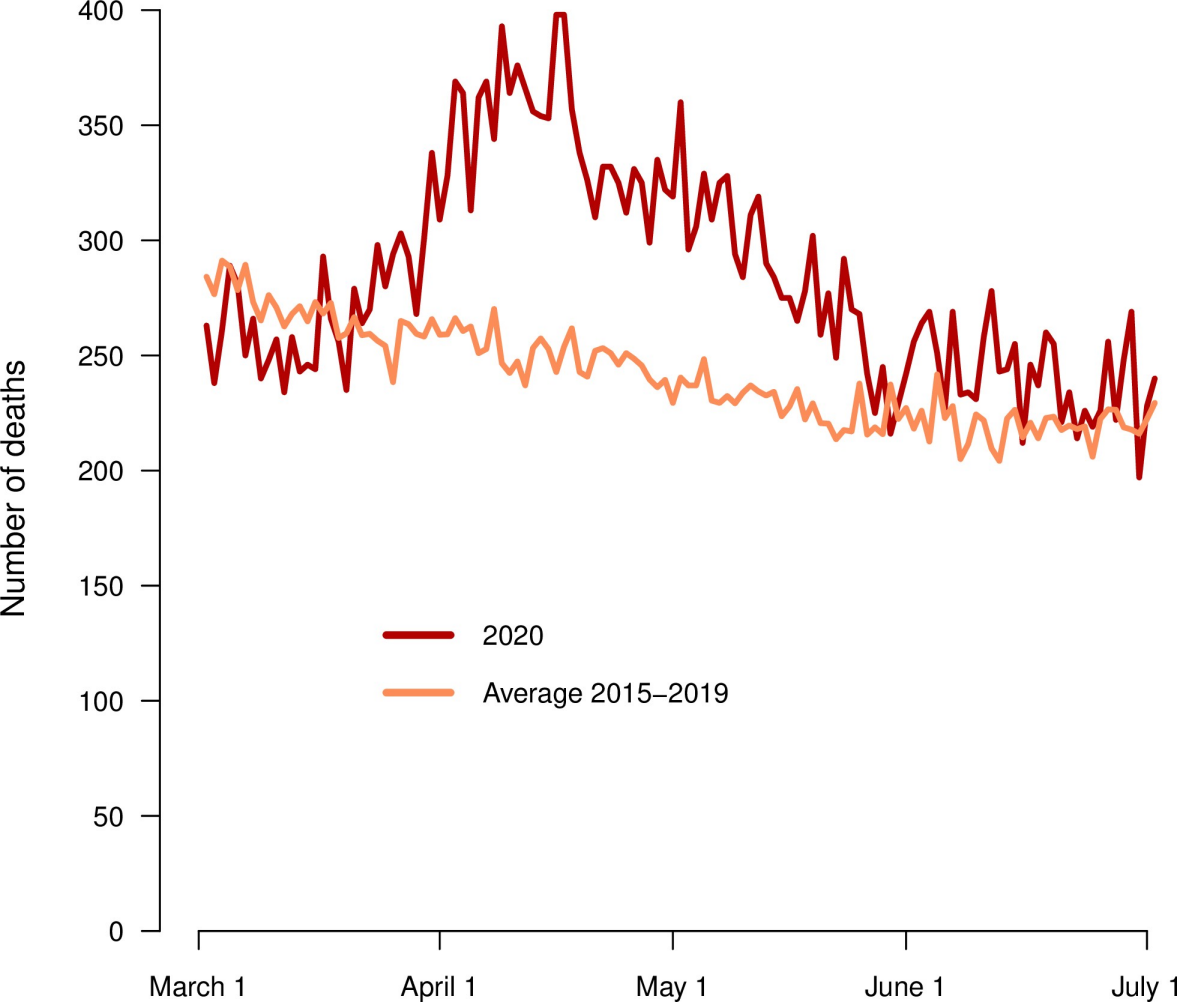
Number of deaths per day in the Swedish general population during the period 1 March to 30 June 2020. The death rate is compared to the mean for corresponding months in 2015–2019.

**Fig 2:**
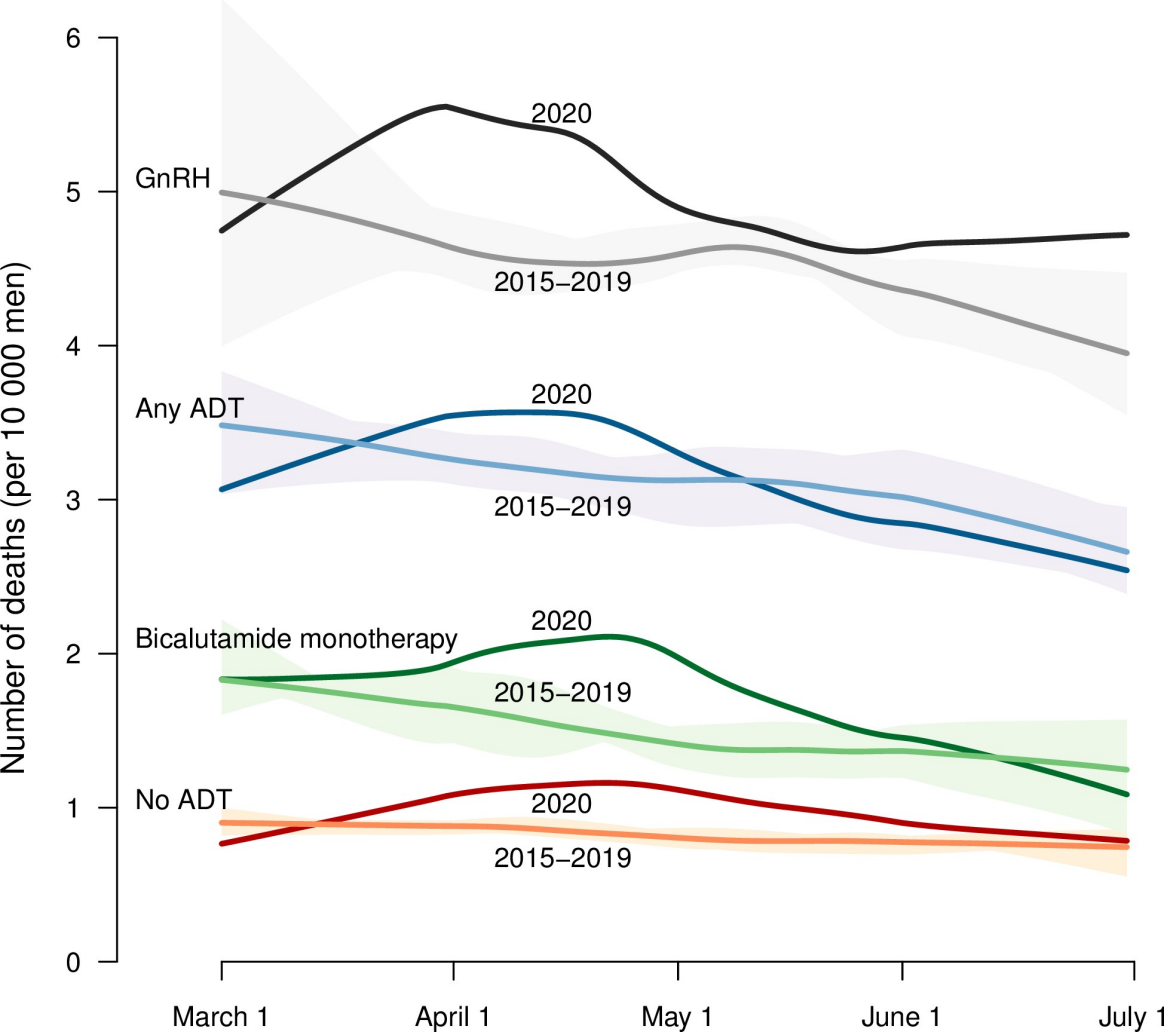
All-cause mortality in men with prostate cancer in March to June 2020 compared to the mean for corresponding months in 2015–2019. Data are presented for men not on androgen deprivation therapy (ADT), men on any ADT, men on bicalutamide monotherapy, and men on GnRH, including GnRH agonists and men who underwent bilateral orchidectomy. The number of deaths by calendar day per 10,000 men with prostate cancer at risk was plotted using loess smoothing. The shaded areas illustrate the range of mortality rates for the separate years 2015 to 2019.

There was a higher number of deaths from all causes in the general Swedish population during the period 1 March to 30 June 2020 compared to the mean for corresponding periods in the previous five years (**Fig 1**). In total, 9,822 men with prostate cancer died during March-June the years 2015–2020, of whom 5,034 men were on ADT and 4,788 men were not on ADT (**Fig 2 and Table 2**). The increase in mortality in March-June 2020 was slightly higher in men on GnRH vs men on bicalutamide. There were large differences in mortality rate according to the type of ADT, but the difference in relative mortality rate comparing the year 2020 to the previous five years within exposure groups was small.

**Table 2:** Association between exposure to GnRH agonists, bicalutamide monotherapy, or any androgen deprivation therapy (ADT) and all-cause mortality in men with prostate cancer in PCBaSe RAPID 2019.

	No ADT		ADT					
			GnRH^a^		Bicalutamide monotherapy		Any ADT	
	March-June 2015–2019	March-June 2020	March-June 2015–2019	March-June 2020	March-June 2015–2019	March-June 2020	March-June 2015–2019	March-June 2020
**Number of deaths**	3,724	1,044	3,066	629	1,055	284	4,121	913
**Observed person-time**, 1,000 person-years	123.5	28.8	18.1	3.3	17.6	4.4	35.7	7.7
**Mortality rate**, per 1,000 person-years	30.2	36.2	169.0	188.7	59.9	64.9	115.3	118.4
**Mortality rate ratio**, year 2020 vs. 2015–2019	1.20		1.12		1.08		1.03	
**Crude relative mortality rate ratio**	Reference		0.93 (0.83–1.04)		0.90 (0.78–1.05)		0.86 (0.78–0.95)	
**Adjusted**^b^ **relative mortality rate ratio**	Reference		**1.00** (0.89–1.12)		**0.97** (0.84–1.12)		**0.96** (0.87–1.06)	

^**a**^ GnRH includes men on GnRH agonists and men who underwent bilateral orchidectomy.

^b^ Adjustment was made for: Age at death in categories, county of residence, prostate cancer risk category, curative treatment, Charlson Comorbidity Index, Drug Comorbidity Index, calendar year, and day in the study period (linear).

Crude comparisons indicated a 7–14% lower excess mortality in men on any ADT compared to men not on ADT and adjustment for potential confounders attenuated these estimates. The crude relative mortality rate ratio comparing the increase in March-June 2020 vs the same period in 2015–2019 for men on any ADT compared to men not on ADT was 0.86 (95% confidence interval (CI) 0.78 to 0.95). This difference was attenuated after adjustment for covariates to 0.96 (95% CI 0.87 to 1.06) (**Table 2**).

When restricting the analysis to the regions with the highest incidence of COVID-19, the results were essentially identical as in the main analysis (**Table 3**). In the analyses restricted to the time period between 2 April to 10 June when mortality in 2020 was increased >30% compared to previous years, men on GnRH had an adjusted relative mortality rate ratio of 0.89 (95% CI 0.77 to 1.03) and men on bicalutamide monotherapy 0.94 (95% CI 0.78 to 1.14).

**Table 3:** Association between exposure to GnRH agonists, bicalutamide monotherapy, or any androgen deprivation therapy (ADT) and all-cause mortality in men with prostate cancer in PCBaSe.

	No ADT		ADT					
			GnRH^a^		Bicalutamide monotherapy		Any ADT	
	March-June 2015–2019	March-June 2020	March-June 2015–2019	March-June 2020	March-June 2015–2019	March-June 2020	March-June 2015–2019	March-June 2020
	**Restricted to the ten Swedish health-care regions with highest incidence of COVID-19**							
**Number of deaths**	2,008	566	1,611	363	596	164	2,207	527
**Observed person-time**, 1,000 person-years	67.3	15.6	9.4	1.8	9.3	2.3	18.6	4.1
**Mortality rate**, per 1,000 person-years	29.8	36.3	172.2	203.7	64.3	70.9	118.6	128.7
**Mortality rate ratio**, 2020 vs. 2015–2019	1.22		1.18		1.10		1.09	
**Crude relative mortality rate ratio**	Reference		0.97 (0.84–1.13)		0.91 (0.74–1.10)		0.89 (0.78–1.02)	
**Adjusted**^b^ **relative mortality rate ratio**	Reference		**1.04** (0.89–1.20)		**0.98** (0.80–1.19)		**0.99** (0.87–1.13)	
	**Restricted to the time period 2 April to 10 June when there was >30% increase in mortality in 2020 compared to 2015–2019**							
**Number of deaths**	2,099	649	1,735	359	589	171	2,324	530
**Observed person-time per** 1000 person-years	70.8	16.5	10.3	1.9	10.1	2.5	20.5	4.5
**Mortality rate** (per 1000 person-years)	29.7	39.4	167.7	184.4	58.3	68.2	113.6	119.0
**Mortality rate ratio** 2020 vs 2015–2019	1.33		1.10		1.17		1.05	
**Crude relative mortality rate ratio**	Reference		0.83 (0.72–0.96)		0.88 (0.73–1.07)		0.79 (0.69–0.90)	
**Adjusted**^b^ **relative mortality rate ratio**	Reference		**0.89** (0.77–1.03)		**0.94** (0.78–1.14)		**0.88** (0.77–1.00)	

^a^ GnRH includes men on GnRH agonists and men who underwent bilateral orchidectomy

^b^ Adjustment was made for: Age at death in categories, county of residence, prostate cancer risk category, curative treatment, Charlson Comorbidity Index, Drug Comorbidity Index, calendar year, and day in the study period (linear).

Analyses restricted to regions with highest incidence of COVID-19 by end of June 2020, and to the time period 2 April to 10 June 2020 when there was a peak in excess mortality.

## Discussion

In this population-based cohort of men with prostate cancer, we found no difference in relative excess mortality in men with prostate cancer on GnRH agonists or bicalutamide monotherapy vs those not on ADT during the peak of the first wave of the COVID-19 pandemic in March-June 2020, compared to the average from corresponding months in 2015–2019. There was only a weak reduction in one of the restricted subgroup analyses, seen in both exposure groups. Considering the mechanistic hypothesis an effect would mainly be expected from exposure to GnRH agonists but no notable difference was seen in the outcome compared to bicalutamide monotherapy. Thus, there was no clear support for the hypothesis that ADT mitigates the course of COVID-19.

The hypothesis that ADT mitigates the course of COVID-19 has been corroborated by *in vitro* studies showing that androgens facilitate cellular uptake of the SARS-CoV-2 virus. In humans, men have a 50% higher risk of infection and hospitalization, critical care admission, and death from COVID-19 compared with women [32–35]. This increase in risk remained after adjustment for age, ethnicity, socio-economic factors, smoking, and concomitant and previous diseases [1].

It is therefore of interest to investigate if ADT mitigates COVID-19. An early study suggested a lower risk of COVID-19 in men with prostate cancer on ADT compared with men not on ADT [10]. Similarly, in a small observational study of 22 men on ADT these men had lower rates of hospitalization, supplemental oxygen requirements, endotracheal intubation, and mortality than 36 control men not on ADT [36]. In contrast, other pilot studies have reported no protective effect with ADT [11, 12, 37, 38]. However, all of these studies were severely hampered by their small number of exposed men and restricted adjustment for putative confounding factors.

In our main analysis of almost 10,000 men with prostate cancer who died during the study period, and of whom half were on ADT, there was no difference in excess mortality in men on any type of ADT vs men not on ADT. In a restricted analysis of ten regions with the highest COVID-19 burden the results were similar to that in the full study group. In a analysis restricted to the period with the highest mortality, we observed a 12% decrease in excess mortality in men on ADT.

Despite our efforts to adjust for putative confounders, there was likely residual confounding. One way to assess confounding is to use the E-value which is defined as the weakest association that an unmeasured confounder would need to have with both exposure and outcome to fully eliminate an association [39]. The E-value was 1.53 for a relative mortality rate ratio of 0.88, which was the most extreme point estimate in all our analyses, including restricted subgroup analyses. An imbalance of exposure to ADT related to prostate cancer risk category [40, 41] as well as an association of risk category with mortality [42] of that magnitude is possible. The finding of no association in the main analysis and a weak association in only one out of six subgroup analyses, likely biased by residual confounding, gives no clear support that any type of ADT mitigates COVID-19.

While there is a hypothetical mechanism for ADT to be potentially beneficial in the early phase of COVID-19, there are also suggestions and preliminary data that ADT could be detrimental in later stages of severe COVID-19, since it could exacerbate or activate an excessive and harmful inflammatory response [43]. It has even been hypothesized that testosterone replacement therapy could be beneficial [44].

There are some other mechanisms, unrelated to suppression of androgen signaling, for an apparent impact of ADT on incidence and severity of COVID-19. For instance, men with advanced prostate cancer on ADT are, compared to men with less advanced cancer who are not on ADT, more likely to impose stricter protective measures such as physical distancing, self-isolation, strict hand hygiene, and wearing a face mask.

A limitation of our study is that the excess in all-cause mortality we observed may not be exclusively caused by COVID-19. The increased mortality during this pandemic has also to some part been attributed to reluctance to seek medical attention for other serious conditions [45]. In a recent study based on data from 29 high-income countries the difference between reported COVID-19 deaths and excess mortality in Sweden was among low, suggesting a high rate of correct adjudication of cause of death in Sweden [46]. Another limitation of our study is the lack of measurements to verify castrate testosterone levels. We found, however, high adherence to GnRH in a recent study in PCBaSe [23] and previous studies have shown that a large proportion of men on GnRH have castrate levels [47]. It would also have been of interest to study men with castration-resistant prostate cancer but we could not identify these men with the data available. Finally, it is unknown if the results from this observational study of old men with prostate cancer on ADT is applicable to the populations in current clinical trials employing short-term ADT in mostly middle-aged men diagnosed with COVID-19.

The strengths of this study are that it was based on a national, population-based cohort, with almost 10,000 deaths and that 5,000 of the deaths were among men on ADT, making it substantially larger than previous human studies combined. The study was performed in a national population of over 10 million people, with high mortality from COVID-19 in the spring of 2020. Furthermore, the study was based on a clinical cancer register and other health care registers with virtually complete capture of comprehensive data with known high quality [14, 48, 49].

In conclusion, there was no difference in relative excess mortality in men with prostate cancer on ADT vs those not on ADT during the peak of the first wave of the COVID-19 pandemic in March-June 2020 compared to the average from corresponding time periods in previous years. Thus, we found no clear support for the hypothesis that androgen deprivation therapy mitigates the disease course of COVID-19.

## Supporting information

S1 Fig Cumulative number of reported cases. The cumulative number of reported cases (per 100,000 population) of SARS-CoV-2 infection to the Public Health Agency of Sweden by the 28 June 2020. The ten regions with the highest cumulative number of cases were used in a subgroup analysis.
(PDF)
