# Supplementary material for: Androgen deprivation therapy and excess mortality in men with prostate cancer during the initial phase of the COVID-19 pandemic
Source: PLoS One. 2021 Oct 7;16(10):e0255966. doi: 10.1371/journal.pone.0255966 (PMC8496782; doi:10.1371/journal.pone.0255966)

Online-only supplementary material

S1 Fig. Cumulative number of reported cases (per 100,000 population) of SARS-CoV-2 infection to the Public Health Agency of Sweden by the 28 June 2020. The ten regions with the highest cumulative number of cases were used in a subgroup analysis.

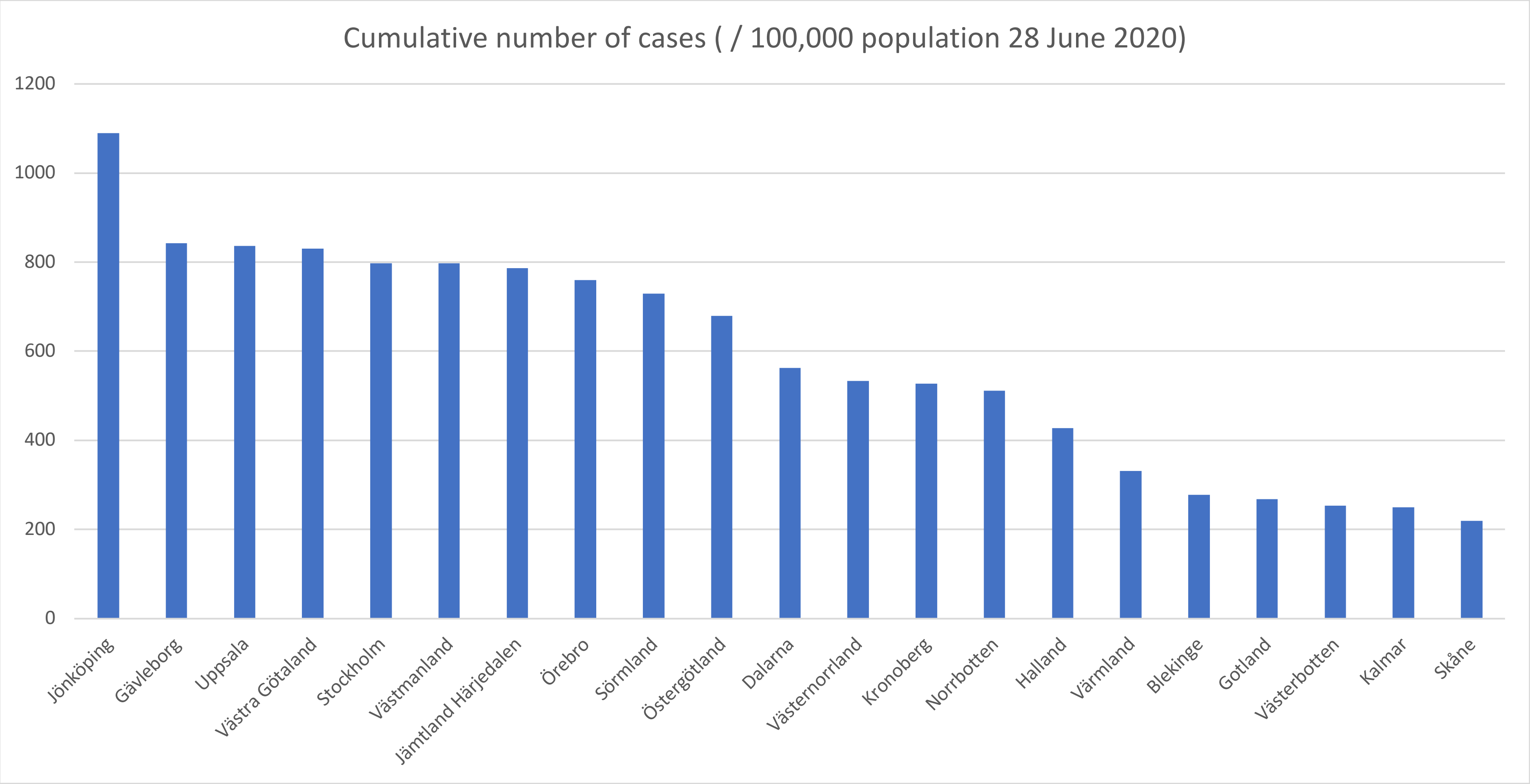

Supplement: S1 Fig — The cumulative number of reported cases (per 100,000 population) of SARS-CoV-2 infection to the Public Health Agency of Sweden by the 28 June 2020. The ten regions with the highest cumulative number of cases were used in a subgroup analysis. (PDF) [file pone.0255966.s001.pdf]
